# P-1256. Pharmacokinetic/Pharmacodynamic (PK/PD) Target Attainment Analyses for Aztreonam-Avibactam Dosing Regimens

**DOI:** 10.1093/ofid/ofae631.1438

**Published:** 2025-01-29

**Authors:** Susan R Raber, Rujia Xie, Elena Soto, Heidi Leister-Tebbe

**Affiliations:** Pfizer, San Diego, California; Pfizer Inc., Singapore, Not Applicable, Singapore; Pfizer R&D UK Ltd., Canterbury, Kent, UK, Canterbury, Kent, England, United Kingdom; Pfizer Inc, Collegeville, Pennsylvania

## Abstract

**Background:**

Aztreonam-avibactam (ATM-AVI) is a fixed-ratio β-lactam/β-lactamase inhibitor recently approved in Europe for adults with serious Gram-negative infections. Prior to commercial availability of ATM-AVI, doses for ceftazidime-avibactam (CAZ-AVI) + ATM have been proposed.^1^ We evaluated probability of joint PK/PD target attainment (PTA) for ATM-AVI and CAZ-AVI + ATM in adults with complicated intra-abdominal infection (cIAI).

**Figure:**
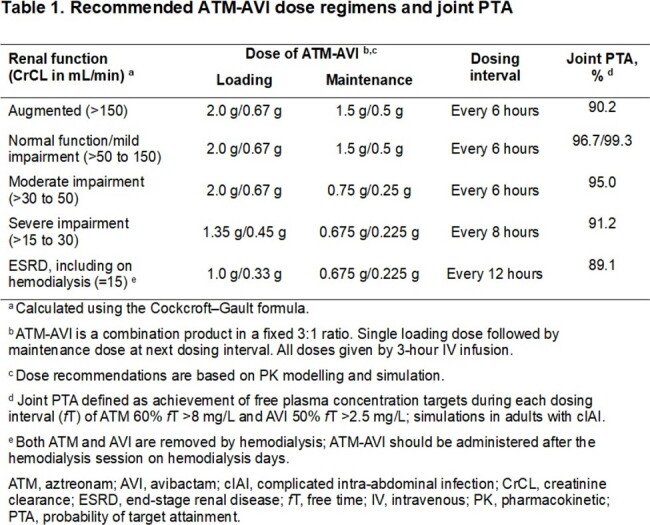

**Methods:**

A simultaneous population PK model for ATM and AVI was developed using data from the CAZ-AVI and ATM-AVI adult clinical programs, including two ATM-AVI phase 3 trials. The final model was used to simulate exposures and calculate PTA (5000 patients/simulation) for ATM-AVI dose regimens adjusted for renal function, and two proposed CAZ-AVI + ATM dose regimens in cIAI patients with CrCL 80 to ≤150 mL/min. PTA was calculated for the joint PK/PD target of ATM 60% *f*T > 8 mg/L and AVI 50% *f*T > 2.5 mg/L.

**Figure:**
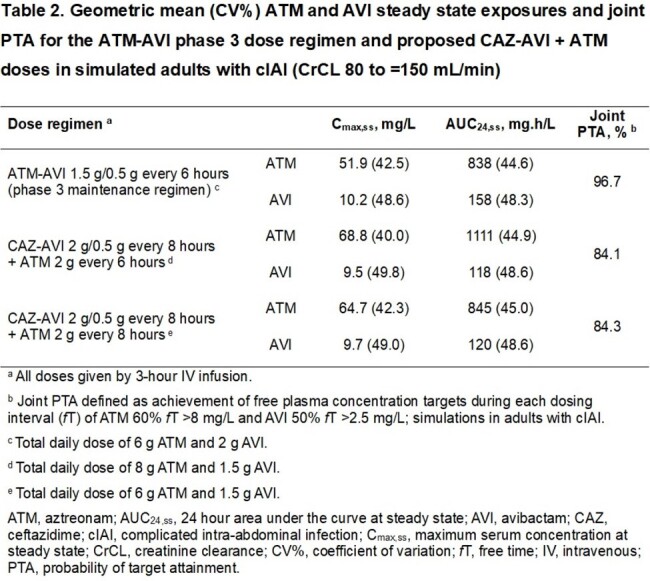

**Results:**

The analysis included 4,914 ATM plasma samples (431 subjects) and 18,222 AVI samples (2,635 subjects). The final model was a two-compartment model for ATM and AVI with zero-order infusion and first-order elimination. Standard allometric exponent functions of body weight on clearances (0.75) and volumes (1) were applied; body surface area-normalized CrCL was used to describe clearance changes during treatment. Infection type was a key covariate on clearance and volume for both drugs. Steady-state exposures for ATM-AVI dose regimens were associated with joint PTA 89 to > 99% across renal function groups (Table 1). CAZ-AVI + ATM dose regimens resulted in similar (or higher) geometric mean ATM C_max,ss_ and AUC_24,ss_ compared with ATM-AVI, but AVI AUC_24,ss_ was approximately 25% lower, and joint PTA was < 85% (Table 2).

**Conclusion:**

ATM-AVI dose regimens achieved high joint PTA across renal function groups. In contrast, joint PTA with proposed CAZ-AVI + ATM dose regimens for normal renal function were < 85% because of insufficient AVI exposures, regardless of the ATM dose (2g q6h or q8h). Joint PTA with both combinations is driven by the AVI plasma PK/PD target and is independent of pathogen MIC up to 8 mg/L.

1. Tamma PD et al. *Clin Infect Di*s 2023: ciad428.

Trial registration: NCT03329092; NCT03580044. Study sponsored by Pfizer.

**Disclosures:**

**Susan R. Raber, PharmD, MPH**, Pfizer: Employee|Pfizer: Stocks/Bonds (Public Company) **Rujia Xie, PhD**, Pfizer: Former employee|Pfizer: Stocks/Bonds (Public Company) **Elena Soto, PhD**, Pfizer: Employee|Pfizer: Stocks/Bonds (Public Company) **Heidi Leister-Tebbe, BSN**, Pfizer: Employee|Pfizer: Stocks/Bonds (Public Company)

